# The effect of cinnamon supplementation on glycemic control in patients with type 2 diabetes or with polycystic ovary syndrome: an umbrella meta-analysis on interventional meta-analyses

**DOI:** 10.1186/s13098-023-01057-2

**Published:** 2023-06-15

**Authors:** Meysam Zarezadeh, Vali Musazadeh, Elaheh Foroumandi, Majid Keramati, Alireza Ostadrahimi, Rania A. Mekary

**Affiliations:** 1 Student Research Committee, Nutrition Research Center, School of Nutrition and Food Sciences, Tabriz University of Medical Sciences, Tabriz, Iran; 2 School of Nutrition and Food Sciences, Tabriz University of Medical Sciences, Tabriz, Iran; 3 Non- Communicable Diseases Research Center, Department of Nutrition and Biochemistry, Faculty Member of Medicine School, Sabzevar University of Medical Sciences, Sabzevar, Iran; 4 Nutrition Research Center, School of Nutrition and Food Sciences, Tabriz University of Medical Sciences, Tabriz, Iran; 5 School of Pharmacy, MCPHS University, Boston, MA 02115 USA

**Keywords:** Cinnamon, Glycemic index, Umbrella meta-analysis, Type 2 diabetes, Polycystic ovary syndrome

## Abstract

**Background:**

Several meta-analyses reported glycemic-lowering effects of cinnamon, while others reported conflicting findings. In the present study, we aimed to perform an umbrella meta-analysis of previous interventional meta-analyses on the effects of cinnamon on glycemic control in patients with type 2 diabetes (T2D) or with polycystic ovary syndrome (PCOS).

**Methods:**

Relevant studies were searched in PubMed, Scopus, EMBASE, Web of Science, and Google Scholar up to June 2022. Meta-analyses of randomized clinical trials (RCTs) investigating the effects of cinnamon on glycemic indices including fasting plasma glucose (FPG), homeostatic model assessment for insulin resistance (HOMA-IR), insulin, and hemoglobin A1C (HbA1c) were included. Random-effects models were used to perform the umbrella meta-analysis and pool the weighted mean difference (WMD) or standardized mean difference (SMD) and their 95% confidence intervals (CI).

**Results:**

Overall, 11 meta-analyses of RCTs were included. Cinnamon supplementation was effective in reducing serum FPG (WMD: -10.93 mg/dL; 95%CI: -16.22, -5.65; SMD: -0.86; 95%CI: -1.19, -0.52), insulin (WMD: -2.01 IU/mL; 95%CI: -3.96, -0.07; SMD: -0.61; 95%CI: -0.93, -0.30), HOMA-IR levels (WMD: -0.61; 95%CI: -0.91, -0.31; SMD: -0.78; 95%CI: -1.26, -0.30), and HbA1c (WMD: -0.10%; 95%CI: -0.17, -0.03).

**Conclusion:**

Cinnamon can be used as an anti-diabetic agent and an add-on treatment to control glycemic indices among patients with T2D or PCOS.

**Supplementary Information:**

The online version contains supplementary material available at 10.1186/s13098-023-01057-2.

## Background

Type 2 diabetes mellitus (T2D), as a metabolic disorder, is a public health problem worldwide. It is predicted that the number of diabetic patients will reach 600 million by 2035 [1]. High prevalence of diabetes is related to increasing incidence of other disorders, reducing the quality of life, and increasing health care costs in every society [2]. Moreover, polycystic ovary syndrome (PCOS) is the most common endocrine disturbance among women of reproductive age [3] and is associated with an increased risk of T2D [4]. The prevention and reduction of diabetes complications will be achieved by means of T2D control [5]. Improving anthropometric indices including body weight, waist circumference, and body composition along with lifestyle modifications and drug therapy are the main treatments for T2D [6, 7]. There are several treatments and anti-diabetic drugs; however, most of them have substantial side-effects. Consequently, the tendency of people to alternative and complementary therapies have significantly increased [8]. In this regard, the anti-diabetic effects of many medicinal herbs have been studied so far [9–12].

Cinnamomum (cinnamon), is a dietary component and a traditional herbal medicine [1, 13, 14]. There is evidence for the beneficial health effects of cinnamon such as anti-diabetic, lipid-lowering, anti-tumor, and antioxidant properties [2]. Moreover, several studies reported the hypoglycemic properties of cinnamon [15–17]. Based on several studies, oral cinnamon supplementation in PCOS patients led to weight loss [18, 19]. It was also shown to be beneficial for regulating the menstrual cycle and improving gynecological, respiratory, and digestive disorders [20, 21]. While some meta-analyses showed a beneficial effect of cinnamon on glycemic indices [16, 22–25], other meta-analyses did not report a significant effect [14, 26, 27]. Additinally, the high heterogeneity in the results of the previously conducted meta-analyses led to an uncertain conclusion on the effects of cinnamon in patients with T2D or PCOS. Therefore, the present umbrella meta-analysis aimed to examine the effects of cinnamon supplementation on serum levels of fasting plasma glucose (FPG), homeostatic model assessment for insulin resistance (HOMA-IR), insulin, and hemoglobin A1c (HbA1c) by performing a meta-analysis on the previously published meta-analyses of randomized clinical trials (RCTs) in patients with T2D or PCOS.

## Methods

### Search strategy and study selection

The scientific international databases including PubMed, Scopus, EMBASE, Web of Science, and Google Scholar were searched for relevant studies published up to June 2022. The search strategy was developed using the appropriate MeSH and title/abstract keywords (**Supplementary Table 1**). To increase the sensitivity of the search strategy, the wild-card term‘‘*’’ was used. Two independent reviewers (VM and MK) screened the articles based on the eligibility criteria. In the first step, the title and abstract of the articles were reviewed. Secondly, the full-texts of related articles were assessed to ascertain the suitability of the study to include in the umbrella meta-analysis. Any disagreement was resolved through the judgment of the third author (MZ).

### Inclusion and exclusion criteria

Meta-analyses of randomized clinical trials investigating the effects of cinnamon supplementation on glycemic indices (FPG, HOMA-IR, insulin, and HbA1c) were included in the current umbrella meta-analysis, if such respective pooled effect sizes and their corresponding confidence intervals (CIs) were reported. In vitro, in vivo, and ex-vivo studies, case reports, observational studies, and quasi-experimental studies were excluded from this umbrella meta-analysis. Only articles in English language were included in the study.

### Risk of bias assessment

The methodological quality assessment of the eligible papers was examined by two reviewers independently (VM, MK), using the AMSTAR questionnaire [28]. The AMSTAR questionnaire contains 11 items revolving around the methodological quality of the systematic reviews and includes answering choices such as “yes, no, cannot answer, or not applicable”. The maximum score is 11. Papers with a score of over seven were considered as high quality.

No small study effect was performed for any of the outcomes as none of them included at least 10 studies [29].

### Data extraction

Publication year, sample size, study location, cinnamon supplementation dosage and duration, effect sizes and their respective CIs for FPG, HOMA-IR, insulin, and HbA1c were extracted from the selected meta-analyses.

### Data synthesis and statistical analysis

Pooled weighted mean differences (WMDs) and standardized mean differences (SMDs) and their respective 95% CIs were extracted to obtain the overall effect sizes for each meta-analysis. Heterogeneity was determined by I^2^ index and Cochrane’s Q test. I^2^ value > 40% or P < 0.1 for the Q-test was considered as a statistically significant between-study heterogeneity [30]. To find probable sources of heterogeneity, subgroup analyses were performed according to study population (T2D, PCOS, and others), gender (women, both) and sample size (≤ 500, ˃500), when data were provided. A one-study removal sensitivity analysis was used to detect the dependency of the overall effect size on a particular meta-analysis. Due to the natural differences between SMD and WMD, the analysis was performed for each separately. The meta-analysis was carried out using Stata, version 1 (Stata Corporation, College Station, TX, US). Unless otherwise specified, a two-sided p-value < 0.05 was considered statistically significant.

## Results

### Study characteristics

**Fig. 1:**
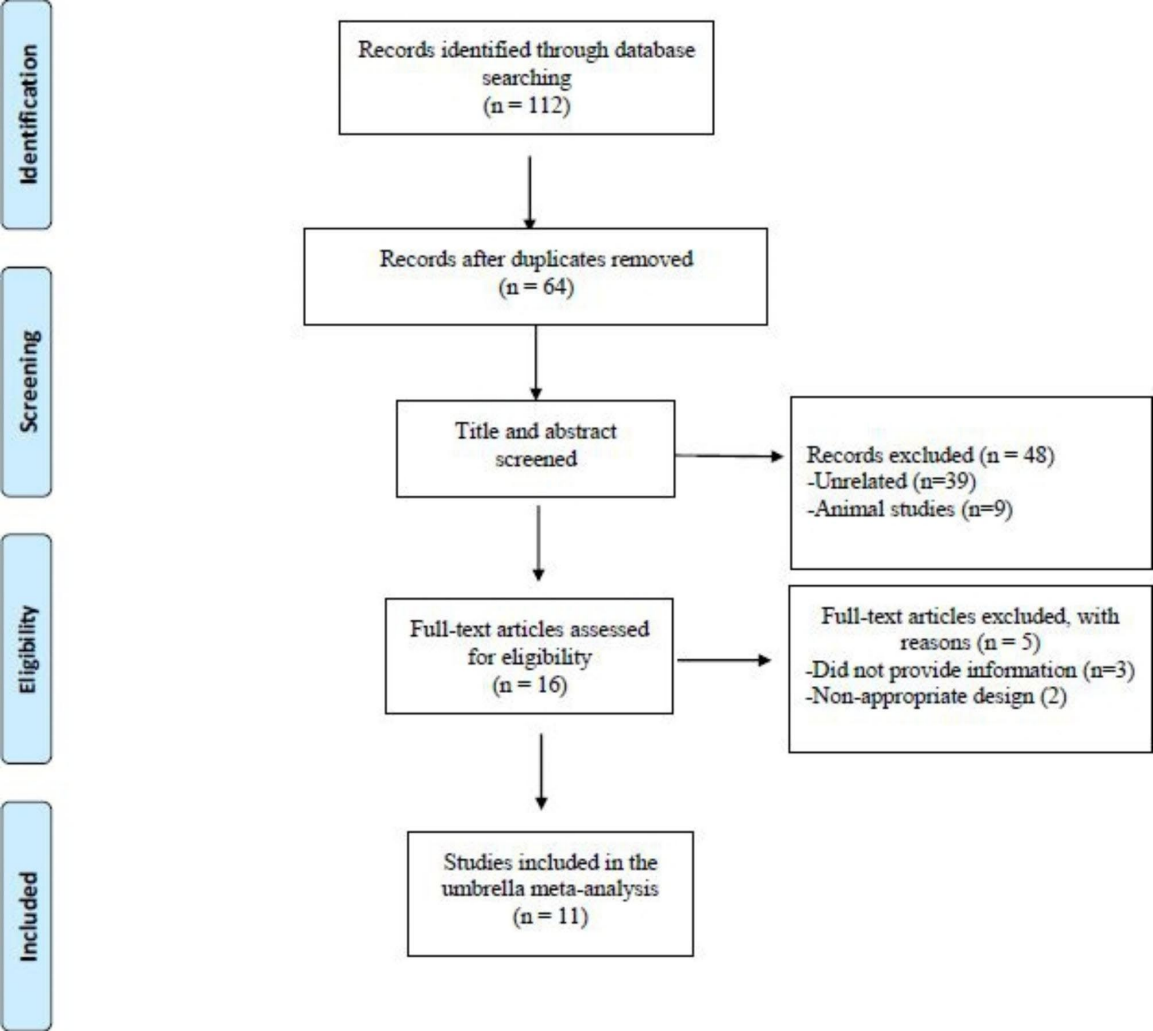
Flow diagram of study selection

Eleven meta-analyses of RCTs (seven on T2D participants, three on women with PCOS, and one on participants with metabolic syndrome) published between 2008 and 2021 were included in the current study. The flow diagram for the selection of the included meta-analyses is presented in Fig. 1. General characteristics of the included studies are summarized in Table 1. Four studies were conducted in Iran [16, 23, 25, 26], three in the USA [14, 20, 31], one in Australia [27], one in Uganda [24], one in Saudi Arabia [32], and another in the UK [22]. The duration of interventions ranged between four and 52 weeks. The dose of cinnamon supplements ranged between 0.12 and 14.4 g/day with a median dose of 3.76 g/day. The Cochrane risk of bias assessment tool was used to assess the quality of the included RCTs in the published meta-analyses [33]. On average, half of the included RCTs was rated as high quality, according to the authors. As for the quality of the meta-analyses included in our study, eight were rated as high quality [16, 22–27, 32], while three were considered to have low quality [14, 20, 31]. Most of the included meta-analyses in this umbrella did not explain the detailed characteristics of the qualified RCTs nor the quality assessment process of their included studies in formulating conclusions; thus, it might affect their overall quality (**Supplementary Table 2**).

**Table 1:** Characteristics of included studies

Citation (First author et al., year)	No. of Studies in Meta-analysis	Location Duration of intervention	No. of Participants in Meta-analysis	Intervention/daily dose	Quality Assessment Scale and Rating	Measured outcomes and Results
Kutbi et al. 2021	23	Saudi Arabia 11	1516 with metabolic diseases	Cinnamon/2.2 g/day	Yes (Cochrane) 23/23 were low	FPG (↓), HOMA (↓), HbA1C(↓), Insulin (↓)
Heshmati et al.2021	5	Iran 8-52wk	289 with PCOS	Cinnamon /0.5–1.5 g/day	Yes (Cochrane) 3/5 were high	FPG(↓), HOMA(↓), insulin(↓)
Heydarpour et al.2020	5	Iran 6-24wk	448 with PCOS	Cinnamon/ 0.336-1.5 g/day	Yes (Cochrane) 3/5 were high	FPG(↓), HOMA (↓), insulin (↓)
Namazi et al.2019	18	Iran 6-17wk	1100 with T2DM	Powder and extract of cinnamon/ 1–6 g/day of powder and 0.12 − 0.5 g/day of extract	Yes (Cochrane) 13/18 were low	FPG (↓), HbA1C (↓)
Deyno et al.2019	16	Uganda 4-16wk	1098 with T2DM	Cassia, aromaticum, zeylanicum and verum/1-14.4 g/day	Yes (Cochrane) 8/16 were high	FPG (↓), HOMA (↓), HbA1C(Ns), Insulin (↓)
Ainehchi et al.2019	16	Iran NR	668 with PCOS	Cinnamon /without restrictions regarding dose	Yes (Cochrane) 7/16 were high	FPG (↓)
Allen et al.2013	10	USA 4-18wk	543 with T2DM	Aqueous cinnamon extract or raw cinnamon powder/0.12-6 g/day	Yes (Cochrane) 5/10 were high	FPG (↓), HbA1C (Ns)
Akilen et al.2012	6	UK 6-16wk	375 with T2DM	Cinnamomum cassia/1–6 g/day	Yes (Cochrane) 2/6 were high	FPG (↑), HbA1C (↑)
Leach et al.2012	6	Australia 4-16wk	577 with T2DM	Cassia, Chinese cinnamon and burmanii/2 g/day	Yes (Cochrane) 3/10 were high	FPG(Ns),HbA1C (Ns),insulin (Ns)
Davis et al.2011	8	USA 4–16 wk	369 with T2DM	1–6 g cinnamon and 250 mg-3 g cinnamon extract	Yes (Cochrane) 3/8 were high	FPG(↓)
Baker et al.2008	4	USA 12	207 with T2DM	Cinnamon/3.5 g/day	NR	FPG (Ns), HbA1C(Ns)

Abbreviations: PCOS, Polycystic Ovary Syndrome; T2DM, type 2 diabetes mellitus;FPG;Fasting blood glucose, HbA1C;Hemoglobin A1C,HOMA; homeostatic model assessment, NS; Not significant, NR; Not reported

### The effects of cinnamon supplementation on FPG levels

**Fig. 2:**
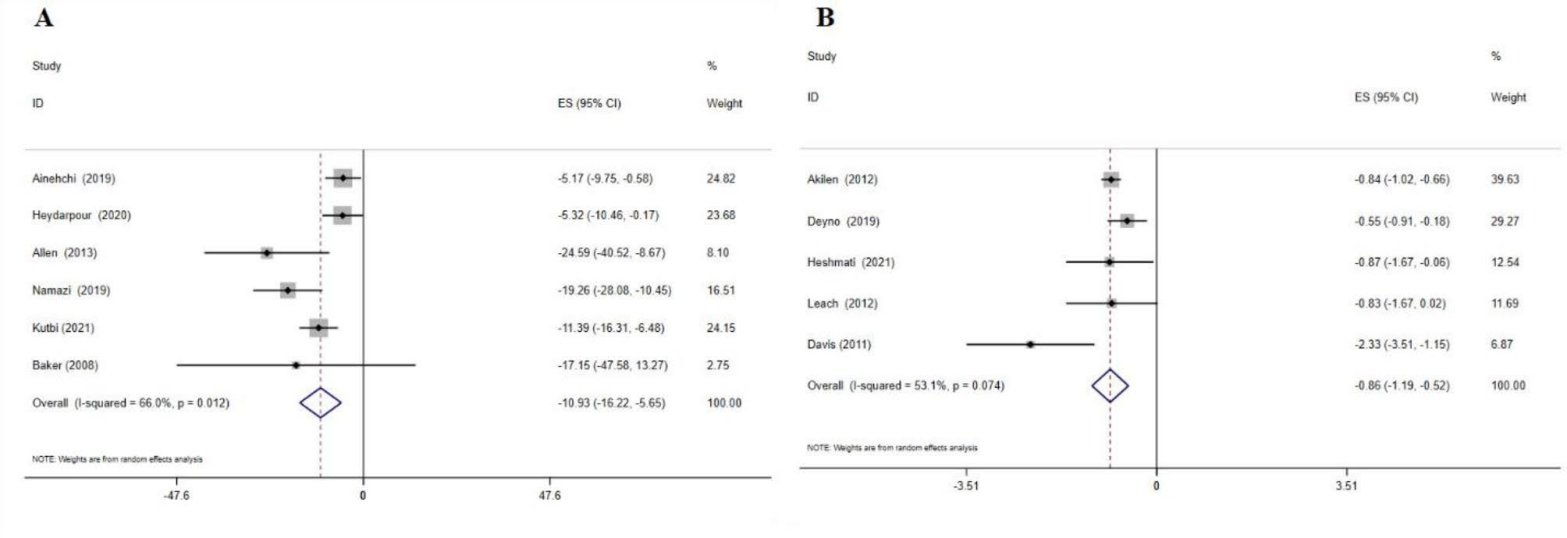
Forest plot detailing effect size and 95% confidence intervals (CIs), the effects of cinnamon supplementation on FPG levels according to WMD analysis(A), and SMD analysis

According to the WMD analysis, the result of combining the data from six meta-analyses showed a significant effect of cinnamon supplementation on FPG reduction (WMD: -10.93 mg/dL; 95% CI: -16.22, -5.65, p = 0.01; six meta-analyses) **(**Fig. 2A**)**. A significant between-study heterogeneity was identified (*I*^*2*^ = 66.0%, p-heterogeneity = 0.01), which was reduced when subgrouping by each of gender and study population **(**Table 2). Based on subgroup analyses, cinnamon supplementation showed a reduction in FPG in all subgroups, with the greatest effect in patients with T2D, in meta-analyses with sample size > 500, and in studies with both gender **(**Table 2). Sensitivity analysis showed that the overall WMD did not depend on any single study. Similarly, the SMD results showed a significant effect of cinnamon supplementation on FPG reduction (SMD: -0.86; 95% CI: -1.19, -0.52, p = 0.01; I^2^ = 53.1%, p-heterogeneity = 0.07; 5 meta-analyses) (Fig. 2B). Cinnamon supplementation led to greater reductions in FPG levels in studies with sample size ≤ 500 (Table 2). Sensitivity analysis indicated the lack of dependence of overall results on one single study.

**Table 2:** Results of subgroup analyses based on various independent variables

Outcomes stratified by different covariates	No. studies	Pooled effect size (95% CI)	P-value	I^2^ (%)	P-heterogeneity
**FPG, Overall (WMD)** **Gender** Women Both **Sample size** > 500 ≤ 500 **Population** PCOS T2DM Other	6 2 4 4 2 2 3 1	-10.9 (-16.2, -5.65) -5.24 (-8.7, -1.81) -15.6 (-21.6, -9.56) -13.0 (-20.1, -5.88) -5.65 (-10.7, -0.58) -5.24 (-8.66, -1.81) -20.3 (-27.8, -12.8) -11.4 (-16.31, -6.47)	< 0.001 0.003 0.000 < 0.001 0.029 0.003 < 0.001 < 0.001	66 0.0 28.4 75.1 0.0 0.0 0.0 -	0.01 0.97 0.24 0.01 0.45 0.97 0.83 -
**FPG, Overall (SMD)** **Sample size** > 500 ≤ 500 **Population** PCOS T2DM	5 2 3 1 4	-0.86 (-1.19, -0.52) -0.59 (-0.92, -0.25) -1.15 (-1.84, -0.46) -0.87 (-1.68, -0.07) -0.88 (-1.28, -0.48)	< 0.001 < 0.001 < 0.001 0.034 < 0.001	53,1 0.0 66.6 - 64.7	0.07 0.54 0.05 - 0.04
**HbA1C, Overall (WMD)** **Sample size** ≥ 1000 < 1000 **Population** T2DM Other	8 3 5 6 2	-0.10 (-0.17, -0.03) -0.16 (-0.25, -0.06) -0.06 (-0.15, 0.03) -0.09 (-0.14, -0.05) 0.01 (-0.51, 0.53)	0.003 < 0.001 0.219 < 0.001 0.962	28.2 0.0 31.9 0.0 85.1	0.20 0.41 0.21 0.71 0.01

Abbreviations: FPG; Fasting plasma glucose, PCOS; Polycystic ovary syndrome, T2DM; Type 2 diabetes mellitus, HbA1C; Hemoglobin A1C, WMD; Weighted mean difference, SMD; Standardized mean difference, MD; Mean difference, CI; Confidence interval; Other: study populations other than T2DM and PCOS

### The effects of cinnamon supplementation on HbA1c levels

**Fig. 3:**
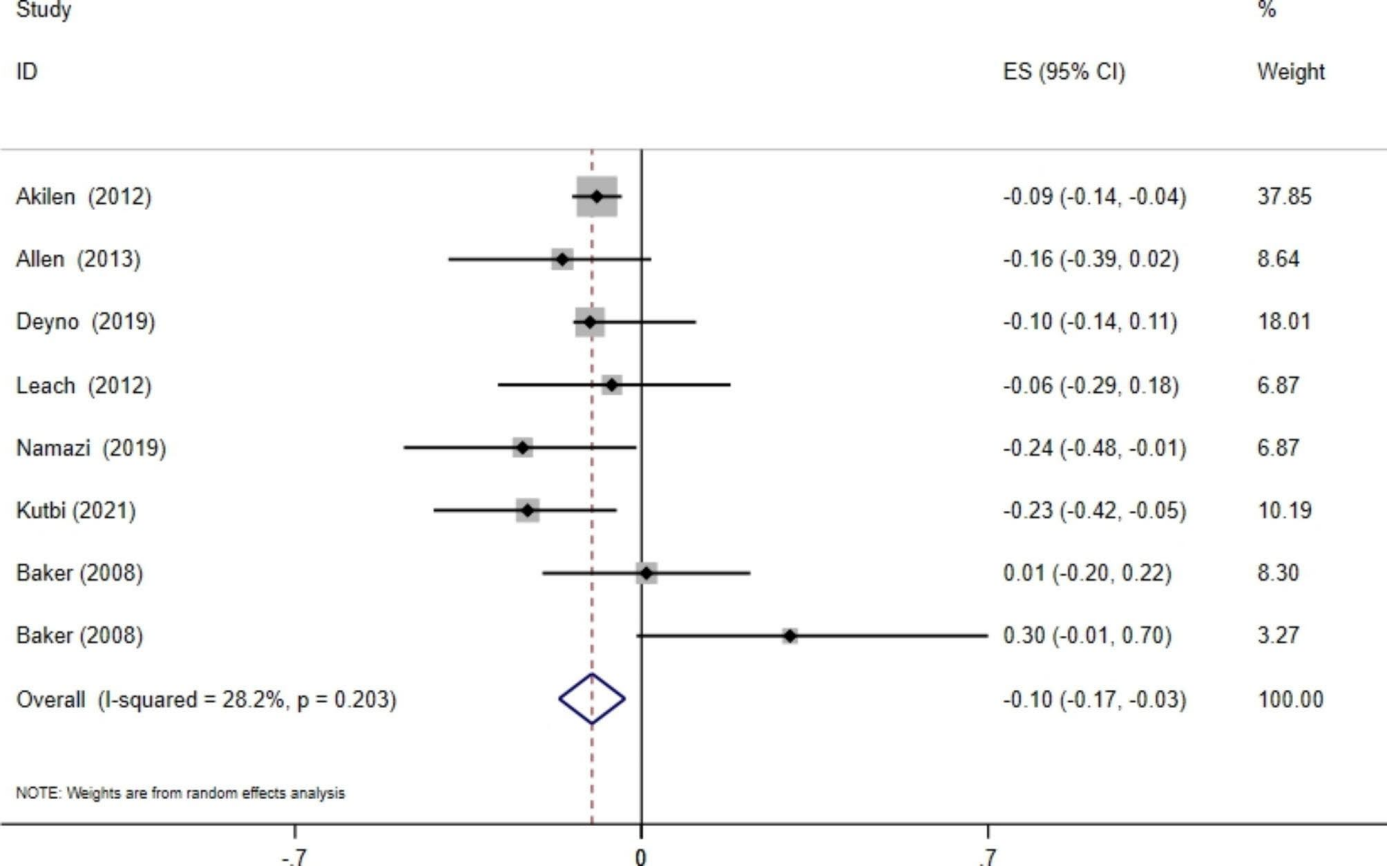
Forest plot detailing mean difference and 95% confidence intervals (CIs) of the effects of cinnamon supplementation on HbA1C levels according to WMD analysis

Combining the data from seven meta-analyses with eight effect sizes indicated a significant effect of cinnamon supplementation on HbA1c levels (WMD: -0.10%; 95% CI: -0.17, -0.03, p = 0.01), without significant between-study heterogeneity (I^2^ = 28.3%, p-heterogeneity = 0.20) (Fig. 3**)**. This was especially seen in studies with sample sizes of ≥ 1000 and in patients with T2D, where the decrease was statistically significant **(**Table 2**)**. Sensitivity analysis indicated no individual study’s impact on the overall effect size.

### The effects of cinnamon supplementation on insulin levels

**Fig. 4:**
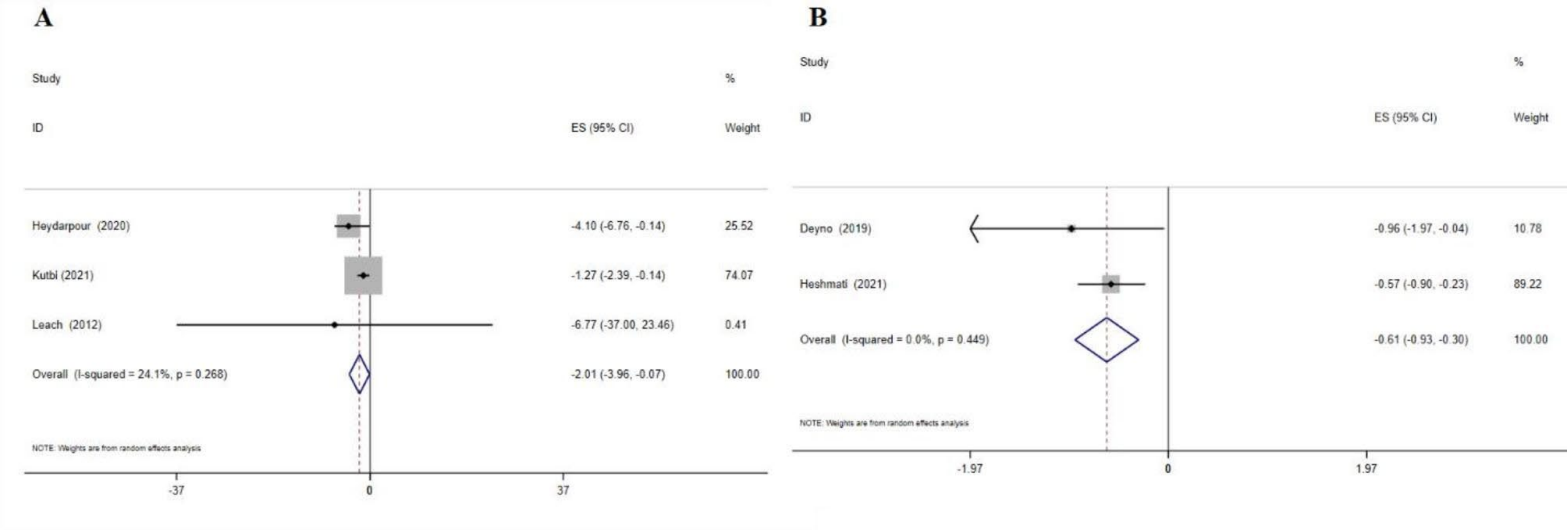
Forest plot detailing effect size and 95% confidence intervals (CIs), the effects of cinnamon supplementation on insulin levels according to WMD analysis(A), and SMD analysis

Cinnamon supplementation showed a considerable decrease in serum insulin levels, according to the WMD analysis (-2.01 IU/mL; 95% CI: -3.96, -0.07; p = 0.04; three meta-analyses) (Fig. 4A**)**, and the SMD analysis (-0.61; 95% CI: -0.93, -0.30; p = 0.01; two meta-analyses) (Fig. 4B). No heterogeneity was observed between studies.

### The effects of cinnamon supplementation on HOMA-IR levels

**Fig. 5:**
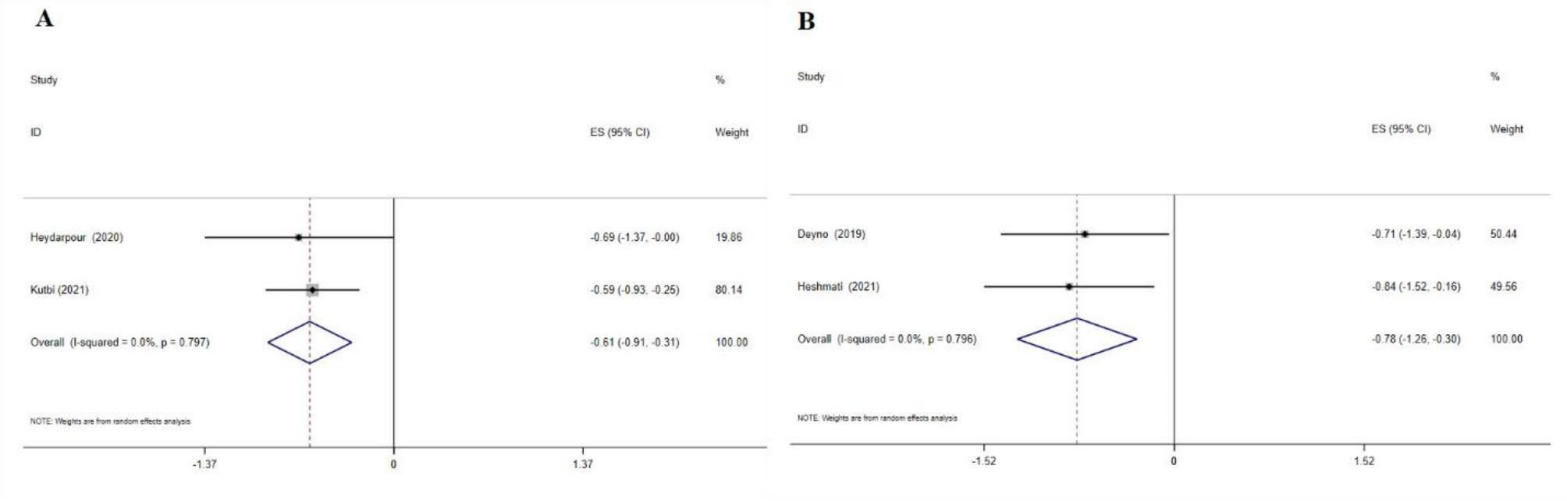
Forest plot detailing effect size and 95% confidence intervals (CIs), the effects of cinnamon supplementation on HOMA-IR levels according to WMD analysis(A), and SMD analysis

Cinnamon supplementation decreased HOMA-IR levels, according to the WMD analysis (-0.61; 95% CI: -0.91, -0.31; p = 0.01; two meta-analyses (Fig. 5A) and SMD analysis (-0.78; 95% CI: -1.26, -0.30; p = 0.01; two meta-analyses) (Fig. 5B). No heterogeneity was observed between studies.

## Discussion

Overall, eleven meta-analyses were included in the current umbrella meta-analysis and the results revealed that cinnamon supplementation significantly reduced serum levels of FPG, insulin, HOMA-IR, and HbA1c in T2D patients and in women with PCOS. For FPG and HbA1c, this reduction was the strongest in T2D participants. Among the included meta-analyses, eight studies (22–28, 35) had high methological qualities. To the best of our knowledge, this is the first umbrella meta-analysis examining the effects of cinnamon supplementation on glycemic indices among patients with T2D or PCOS.

### FPG

The current study demonstrated that cinnamon supplementation significantly reduced serum FPG levels among T2D patients. Recent trials and animal studies releaved that chronic intake of cinnamon promoted satiety and diminished mean food consumption, which contributed to lower FPG and 2-h post-prandial blood glucose concentarions [34, 35]. A recent narrative review reported that consumption of cinnamon along with conventional hypoglycemic medication had a modest benefit on glycemic control [36].

**Fig. 6:**
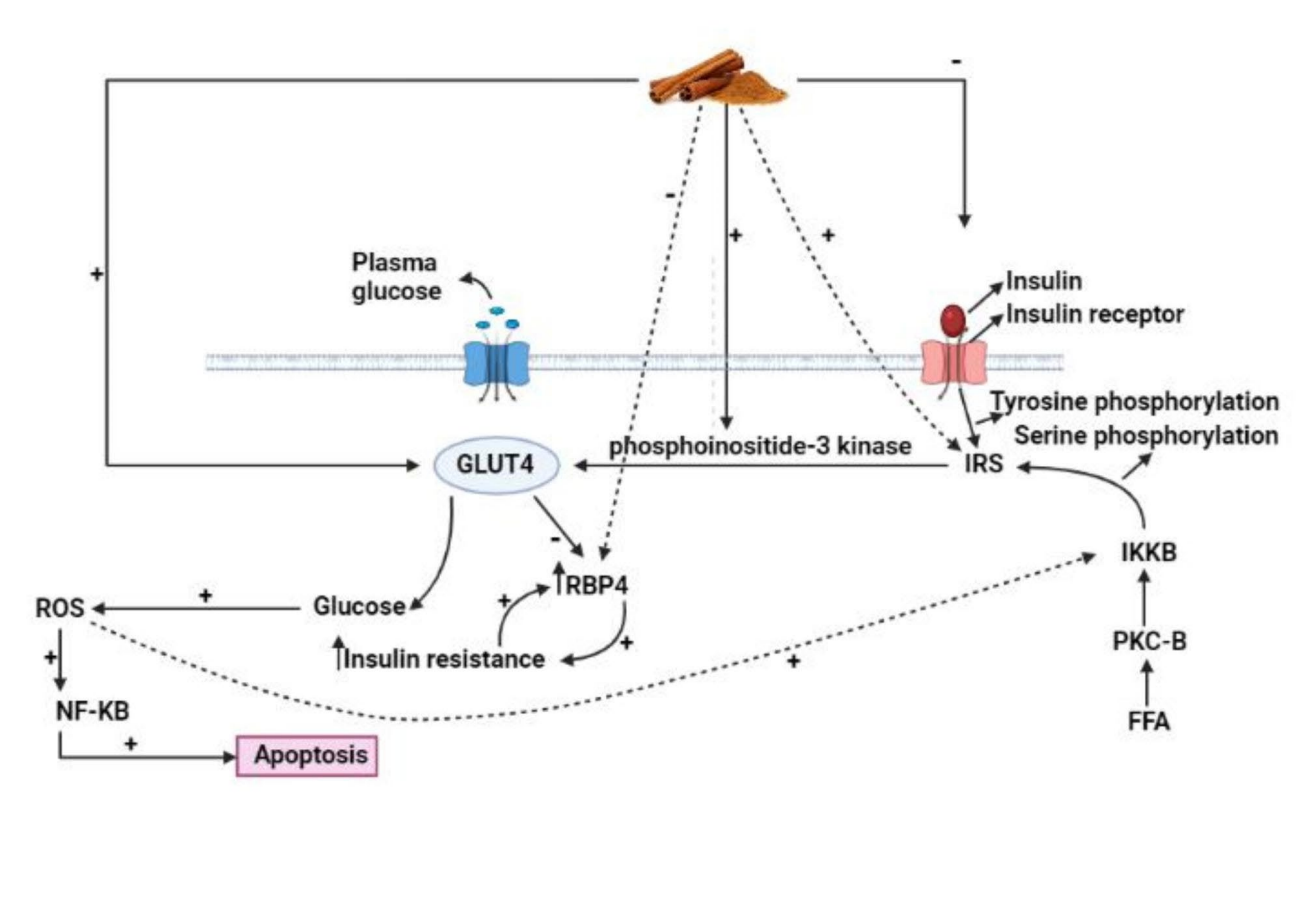
The mechanism of cinnamon intake on glycemic indice

The potential beneficial impact of cinnamon on FPG concentration is due to its effective role in increasing the levels of PI3-kinase and phosphorylated intracellular protein IRS-1, and therefore stimulating the activity of insulin receptors, and increasing cellular glucose uptake [37]. This mechanism is responsible for a dose-dependent reduction in serum insulin levels by cinnamon intake, which was shown in Fig. 6. Furthermore, the bioactive ingredients of cinnamon prepreations may have different results on glycemic control, as it is not clear whether both the extract and powder of cinnamon are equally effective [22]. Taken all together, it is suggested that the ability of cinnamon to lower FPG in T2D and PCOS patients may be due to its polyphenol compounds’ potential to enhance insulin signaling and then potentiate insulin-regulated glucose utilization.

### HOMA-IR levels

In the current study, four meta-analyses have shown a significant reduction of serum HOMA-IR levels by cinnamon supplementation (4, 24, 26, 35). A recent narrative review demonstrated that insulin resistance had a central role in the pathology of PCOS and was prevalent in 70% of PCOS patients. [38]. This puts pressure on the pancreas to produce more insulin, which causes a gradual destruction of beta cells [39], and leads to pre-diabetes and T2D.

Cinnamon can improve HOMA-IR levels through various mechanisms including downregulating insulin signaling in adipocytes [40], inhibiting alpha-amylase action as initial carbohydrate digestion enzyme, activating adenosine mono-phosphate (AMP)-kinase that can regulate the GLUT4, and activating insulin-like growth factor-1 (IGF1) signaling in fibroblasts that can lead to glycemic control [21].

### Insulin resistance

It is suggested that the reduction of insulin resistance among insulin-resitant subjects should be an important goal of disease treatment. The compensatory hyperinsulinemia occurs as the initial response to insulin resistance [39]. The results of the current study has shown that cinnamon can effectively decrease hyperinsulinemia. Concordantly, a previous meta-analysis in 2012 reported beneficial effects of cinnamon on insulin status in both in vitro and in vivo studies on diabetic animals [41]. Notably, in our umbrella meta-analysis, the included studies investigating the effects of cinnamon on insulin levels and HOMA-IR were not sufficient. There is a need for additional studies with focus on the different dosage of cinnamon supplementation, large sample sizes, and longer duration to find an exact conclusion regarding two specific glycemic indices, insulin and HOMA-IR. Moreover, consistently reporting WMD in lieu of SMD is needed as WMD is easier to interpret and assists in generating comparable point estimates that can be pooled across studies.

### HbA1c

The results of current umbrella meta-analysis have shown significant differences in HbA1c levels between cinnamon and placebo groups. Out of the seven included meta-analyses investigating the effect of cinnamon on HbA1c concentations, Namazi et al. [16] and Kutbi et al. [32] had the largest amount of studies and participants compared to the other included meta-analyses, which gave their pooled SMD greater power than the other included meta-analyses. Akilen et al. included patients with baseline HbA1C levels greater than 8% and who were using concomitant hypoglycemic medications, to which cinnamon supplementaion was shown to be statistically significantly beneficial according to their pooled analysis. The other four meta-analyses by Allne et al., Deyno et al., Leach et al., and Baker et al., showed no statistically significant associations between cinnamon supplementation and HbA1c reduction. Neverthelss, the results of current umberalla meta-analysis clearly demonstrated the beneficial effects of cinnamon on HbA1C levels.

This study had some limitations. First, the type of cinnamon and its prepration were different in the included studies, which might have affected the contents of active agents and their hypoglycemic activity. Second, we were not able to conduct subgroup analyses according to supplementation dosage and study duration, as such information was now provided by all of the included studies, which might have contributed to some heterogenity. Third, the potential differences in the baseline values of the different T2D biomarkers across the different meta-analyses might have diluted the effect of cinnamon on glycemic control, as participants with less favorable values would benefit the most. Notwithstanding, our study had some strengths. Conducting the different subgroup analyses helped investigate the effects of cinnamon on glycemic indices more accurately. Furthermore, segragating SMD and WMD in the umbrella meta-analysis made the results more reliable and easier to interpret.

## Conclusion

Cinnamon can be used as an anti-diabetic agent and an add-on treatment to control some glycemic indices among T2D patients and women with PCOS. Future studies with focus on the different dosage of cinnamon supplementation, the bioactive ingredients of cinnamon prepreations, and longer duration are needed to shed light on these specific aspects and their potential role as effect modifiers.

**Table:** 

PICO criteria for the present systematic review and meta-analysis of meta-analysis		
Participants	Adult who were treated with Cinnamon for subjects with type 2 diabetes and Polycystic Ovary Syndrome	
Intervention	Cinnamon OR Cinnamons OR Cinnamomum verum OR Cinnamomum zeylanicum OR Cinnamomum OR Ceylon cinnamon OR cinnamon extract	
Comparator	Placebo or control group	
Outcomes	Glucose OR Sugar OR FBS OR Insulin OR HOMA-IR OR insulin resistance OR QUICKI OR insulin sensitivity OR HbA1c	

## Electronic supplementary material

Below is the link to the electronic supplementary material.

Supplementary Material 1

## Data Availability

Not applicable.

## List of Abbreviations

AMP: Adenosine mono-phosphate
Cis: Confidence intervals
ES: Effect sizes
FPG: Fasting plasma glucose
HbA1c: Hemoglobin A1c
HOMA-IR: Homeostatic model assessment for insulin resistance
IGF1: Insulin-like growth factor-1
PCOS: Polycystic ovary syndrome
RCTs: Randomized clinical trials
SMDs: Standardized mean differences
T2D: Type 2 diabetes mellitus
WMDs: Weighted mean differences
