# Supplementary material for: The effect of cinnamon supplementation on glycemic control in patients with type 2 diabetes or with polycystic ovary syndrome: an umbrella meta-analysis on interventional meta-analyses
Source: Diabetol Metab Syndr. 2023 Jun 15;15:127. doi: 10.1186/s13098-023-01057-2 (PMC10268424; doi:10.1186/s13098-023-01057-2)
Supplement: Supplementary file 1 — Supplementary Material 1 [file 13098_2023_1057_MOESM1_ESM.docx]

| Study | A priori design | selection and data extraction | literature search | publication type | list of studies | characteristics of the included studies | assessed scientific quality | scientific quality formulating conclusions | methods used to combine the findings | assessed  publication  bias | conflict  of interest  stated | Quality score |
| --- | --- | --- | --- | --- | --- | --- | --- | --- | --- | --- | --- | --- |
| Baker et al.2008 | + | - | + | ? | - | - | - | ? | + | + | - | 4 |
| Davis et al.2011 | + | - | ? | ? | - | - | - | ? | + | + | - | 3 |
| leach et al.2012 | ? | + | + | + | + | - | + | + | - | + | - | 7 |
| Akilen et al.2012 | + | - | + | + | + | + | + | ? | + | - | + | 8 |
| Allen et al.2013 | ? | - | - | + | + | - | - | ? | + | + | - | 5 |
| Ainehchi et al.2019 | + | + | + | + | - | - | ? | + | + | - | + | 7 |
| Deyno et al.2019 | + | + | + | + | + | - | + | + | + | + | + | 10 |
| Namazi et al.2019 | + | + | + | + | - | - | + | ? | + | + | + | 8 |
| Heydarpour et al.2020 | + | + | + | + | + | + | + | ? | + | + | + | 10 |
| Heshmati et al.2021 | + | + | + | + | + | - | - | ? | + | + | - | 7 |
| Kutbi et al 2021 | + | + | + | + | + | + | + | ? | + | + | + | 10 |

**Supplemental Table 1**: Results of assess the methodological quality of meta-analysis

The result of assess the methodological quality using AMSTAR: each item for included studies (? ; can't answer; *: Not applicable; - ; means no; +: means yes).
